# Supplementary material for: Content-rich biological network constructed by mining PubMed abstracts
Source: BMC Bioinformatics. 2004 Oct 8;5:147. doi: 10.1186/1471-2105-5-147 (PMC528731; doi:10.1186/1471-2105-5-147)
Supplement: Additional File 2 — The original results of the above study (non-essential files are deleted to keep the file size under the limit set by BMC bioinformatics). [file 1471-2105-5-147-S2.bz2 › chilibotAdditionalFile2/dip30/439ID9507024E10388/html/CCL21_CCR7.html]

 


 **CCL21** and **CCR7** 
  
Found 97 abstracts in PubMed, retrieved 30.  
 

 What does Google say? 
 PDF only 
| .edu only 

---

**Interactive relationship** (e.g. stimulation, inhibition, etc)

**Neutral relationship**- **CCR7**  binds CCL19 and  **CCL21** , causing the highly activated DCs to be trapped in the peripheral tissue site.  Ref: 12414528 Am J Pathol, 2002
- In the present article, we show that maturing Mtb infected DC express high levels of  **CCR7**  and they become responsive to its ligand  **CCL21** .  Ref: 12538673 J Immunol, 2003
- We demonstrate that the CD4 CD25 CD62L splenocytes express  **CCR7**  at high levels and migrate toward secondary lymphoid tissue chemokine  [ **CCL21** ]  and ELC macrophage inflammatory protein 3beta, lymphoid chemokines.  Ref: 12193715 J Immunol, 2002
- Using LC type cells generated in vitro in the presence of transforming growth factor TGF beta1, we further found that tumor necrosis factor TNF alpha, as a prototype proinflammatory factor, and a variety of inflammatory stimuli and bacterial products, increase Langerin expression and Langerin dependent Birbeck granules formation in cell which nevertheless lack costimulatory molecules, DC LAMP CD208 and potent T cell stimulatory activity but express  **CCR7**  and respond to the lymph node homing chemokines CCL19 and  **CCL21** .  Ref: 12186835 J Exp Med, 2002
- Furthermore,  **CCL21**  expression in the inflamed skin was detected in dermal lymphatic endothelial cells and rare  **CCR7**  mature DCs were mostly seen within the lymphatic vessels.  Ref: 12474232 J Pathol, 2003
- In antigen trafficking from the skin, it has been postulated that Langerhans cells dendritic cells are activated after capturing exogenous antigens, up regulate the expression of the chemokine receptor,  **CCR7** , and migrate into lymphoid organs in response to the signaling of a chemokine,  **CCL21** , which is expressed in lymphatic vessels and T cell zone stromal cells.  Ref: 12750361 Int Immunol< MedlineTA>Int Immunol, 2003
- These results identify endothelial  **CCL21**  expression as an important determinant for naive T cell migration to tertiary tissues, and suggest the  **CCL21**   **CCR7**  pathway as a therapeutic target in diseases that are associated with naive T cell recruitment.  Ref: 12707342 J Immunol, 2003

**Non-interactive relationship** (e.g. studied together, co-existance, homology, etc.)

- Using immunohistochemistry, the majority of DC Lamp mature DCs in the T cell area of LNs expressed CCL19 and were surrounded by  **CCR7**  naïve type lymphocytes, while  **CCL21**  was expressed in reticular stromal cells and vascular endothelial cells.  Ref: 12474232 J Pathol, 2003
- We determined the receptor antagonist properties of murine N terminally truncated secondary lymphoid tissue chemokine  [ **CCL21** ]  SLC  [ **CCL21** ]  6Ckine  [ **CCL21** ]  CCR ligand 21 analogs and evaluated the preventive effects of SLC  [ **CCL21** ]  antagonists on chronic graft vs host disease GVHD in a murine model by blocking the homing of donor  **CCR7**  expressing T cells into the recipient s lymphoid organs.  Ref: 12496447 J Immunol, 2003
- Encephalitogenic T cells showed surface expression of  **CCR7**  and the alternative receptor for  **CCL21** , CXCR3.  Ref: 12209625 Eur J Immunol, 2002
- The binding to IP 10 could be inhibited by SLC  [ **CCL21** ]  but not by a non mac25 AGM binding chemokine, EBI1  [ **CCR7** ]  ligand chemokine ELC.  Ref: 12847218 J Immunol, 2003
- The lymphocyte expressed chemokine receptor  **CCR7**  is playing an important role during this process, as its HEV presented ligands CCL19 and  **CCL21**  can trigger rapid integrin activation under flow in addition to inducing a chemotactic response, which may participate in transmigration and or interstitial migration.  Ref: 12393730 Blood, 2003
- This was due to the complete abrogation of upregulation of CC chemokine receptor 7  [ **CCR7** ] , the receptor for MIP3beta SLC  [ **CCL21** ] .  Ref: 12437666 Br J Haematol, 2002
- Upon contact with KpOmpA, human immature DC and macrophages acquire  **CCR7**  expression and responsiveness to  **CCL21** .  Ref: 12548563 Eur J Immunol, 2003
- Costimulation with PGE2 enhanced the expression of the CCL19  **CCL21**  receptor  **CCR7**  on the cell surface of MoDCs when they were matured with soluble CD40 ligand or proinflammatory cytokines, but did not affect  **CCR7**  expression of polyI C stimulated MoDCs.  Ref: 12149218 Blood, 2002
- In the present study, using a murine spleen derived DC line, we analyzed effects of  **CCR7**  ligands, CCL19 and  **CCL21** , on dendritic morphology.  Ref: 12200351 Blood, 2002
- Transcripts for CCL19 and  **CCL21**  and their common receptor  **CCR7**  were induced in the CNS of mice undergoing relapsing remitting and chronic relapsing EAE.  Ref: 12580544 Brain Pathol, 2003
- SLC  [ **CCL21** ]  analogs truncated >4 aa residues from the N terminus showed a loss of chemotaxis and Ca2 influx of  **CCR7**  expressing cells and also inhibited SLC  [ **CCL21** ]  stimulated chemotaxis and SLC  [ **CCL21** ]  induced Ca2 influx completely.  Ref: 12496447 J Immunol, 2003
- In normal skin, on the other hand, no cells immunoreactive for CCL19,  **CCL21** , or  **CCR7**  were found.  Ref: 12474232 J Pathol, 2003
- The CC chemokine receptor CCR 7  [ **CCR7** ]  ligands  **CCL21**  and CCL19 were recently described as essential elements for establishing the microenvironment needed to initiate optimal immune responses in secondary lymphoid tissues.  Ref: 12890630 Virology, 2003
- Here we demonstrate that there is a distinct pathway of antigen trafficking from skin in the steady state that is independent of  **CCL21**   **CCR7**  signaling.  Ref: 12750361 Int Immunol< MedlineTA>Int Immunol, 2003
- The receptor for  **CCL21** ,  **CCR7** , was also found to be highly expressed on the infiltrating T cells, most of which expressed the memory CD45Ro phenotype.  Ref: 12393410 Blood, 2003
- In the present study, we examined the effects of CCL19 and  **CCL21** , two  **CCR7**  ligands, on endocytosis of fluorescein isothiocyanate FITC dextran by murine DCs.  Ref: 12609829 Blood< MedlineTA>Blood, 2003
- RT PCR analysis revealed expression of CCL19,  **CCL21** , and  **CCR7**  at high levels in LNs and at low levels in inflamed skin.  Ref: 12474232 J Pathol, 2003
- SIV infection altered the expression of chemokines CCL19 MIP 3beta,  **CCL21**  6Ckine  [ **CCL21** ] , and CCL20 MIP 3alpha and of chemokine receptors  **CCR7**  and CCR6 that drive DC trafficking.  Ref: 12406887 Blood, 2003
- We show that the  **CCR7**  ligand CC chemokine ligand CCL 21  [ **CCL21** ]  is sufficient for attracting naive T cells into tertiary organs.  Ref: 12707342 J Immunol, 2003
- These cells expressed higher levels of alpha 4 beta 7 integrins than other gammadelta T cell subsets and selectively migrated to the  **CCR7**  ligand secondary lymphoid tissue chemokine  [ **CCL21** ]   **CCL21** .  Ref: 12391210 J Immunol, 2002
- SLC  [ **CCL21** ]  and their shared receptor,  **CCR7** .  Ref: 12474232 J Pathol, 2003
- The increase in intracellular calcium in response to 6Ckine  [ **CCL21** ]  showed that chemokine receptor 7  [ **CCR7** ]  expression was functional.  Ref: 12682236 J Immunol, 2003
